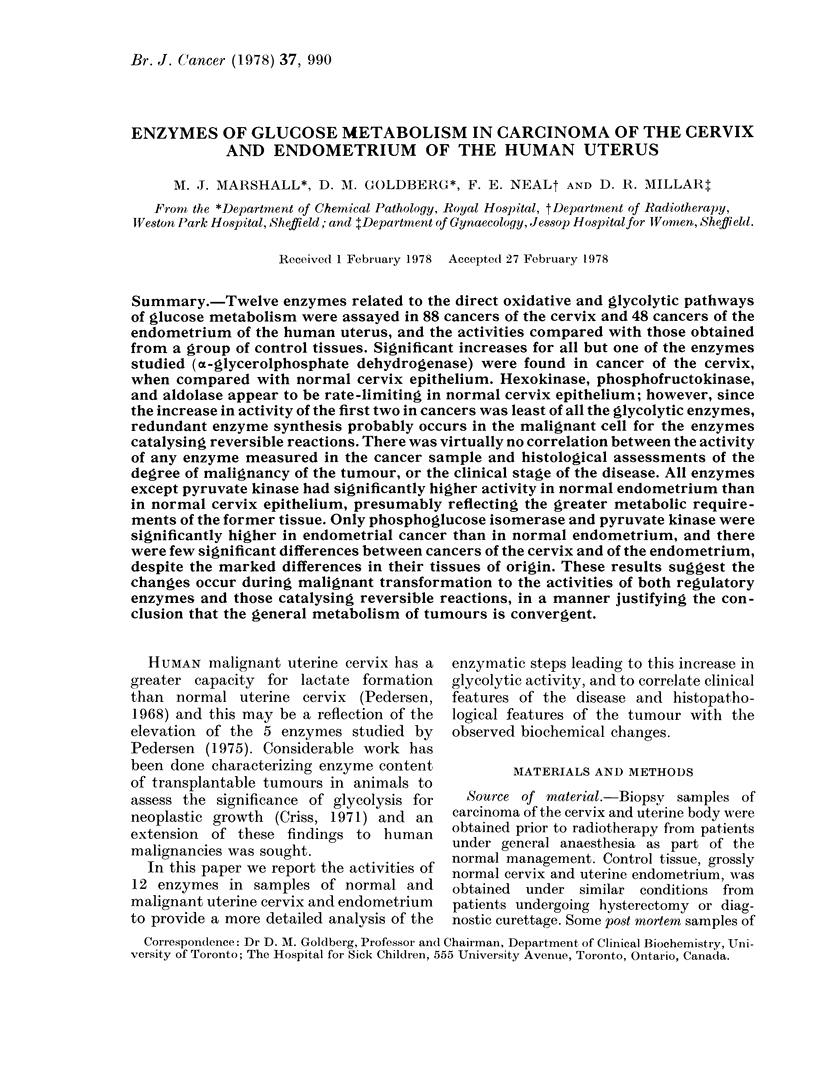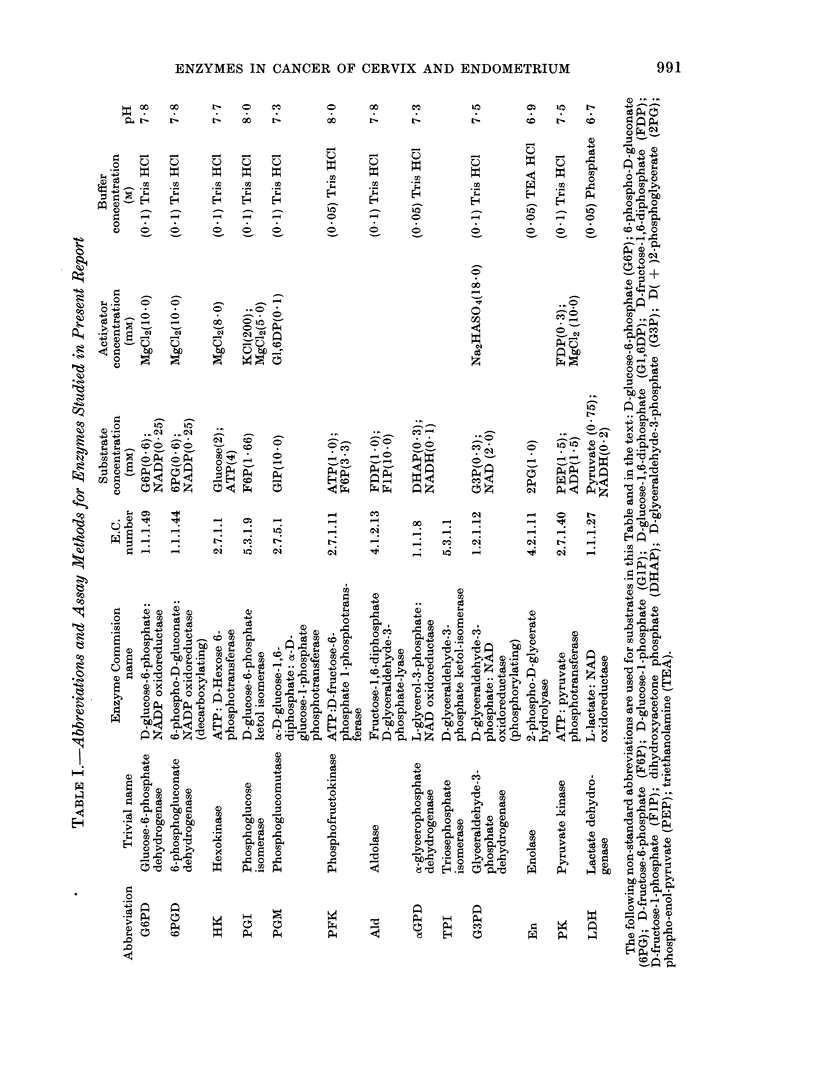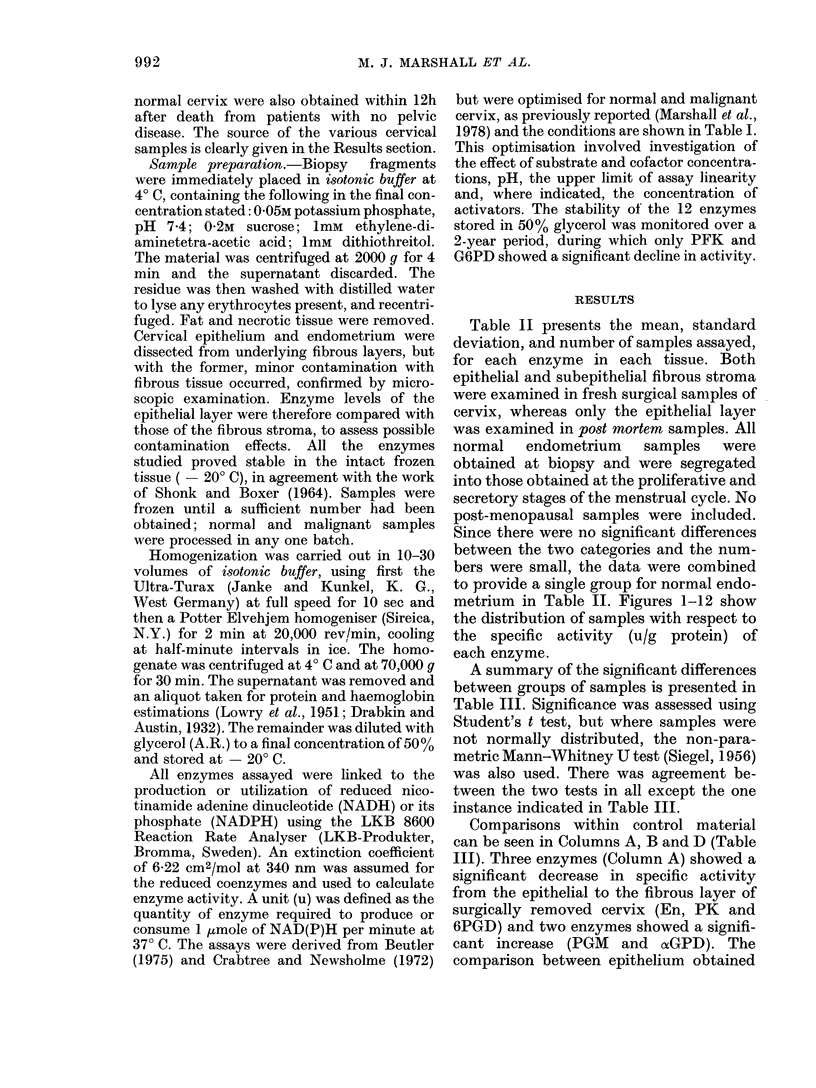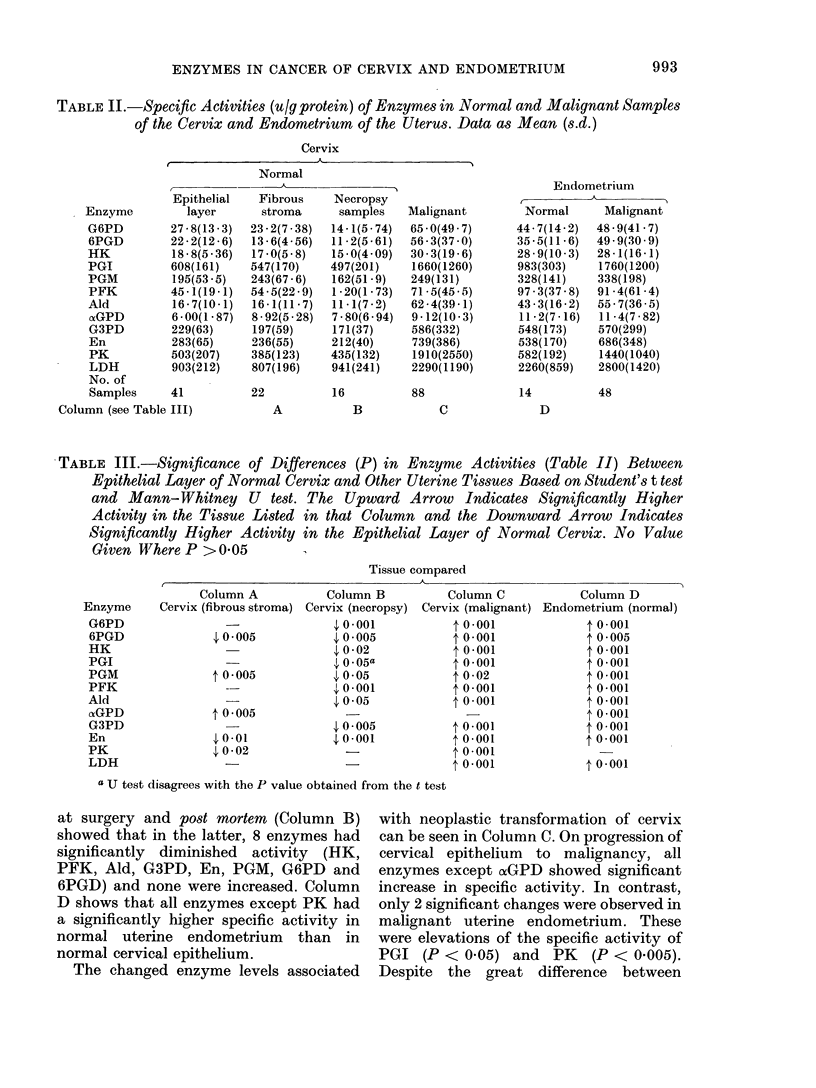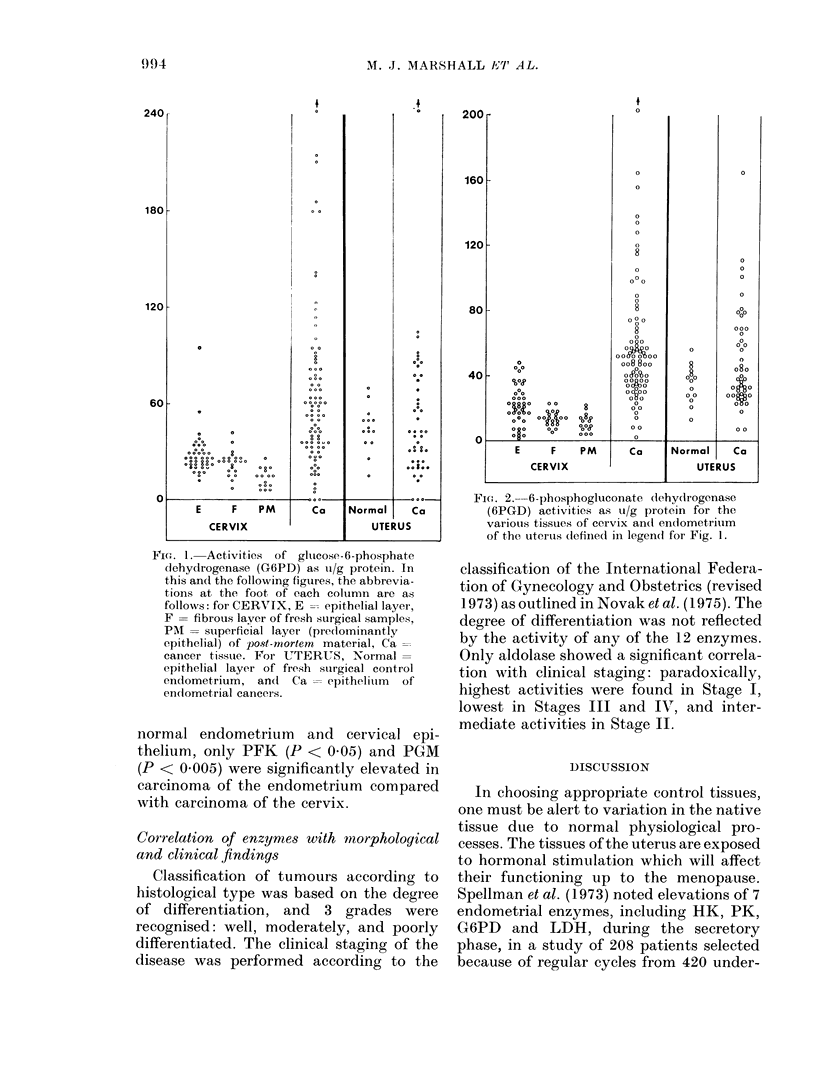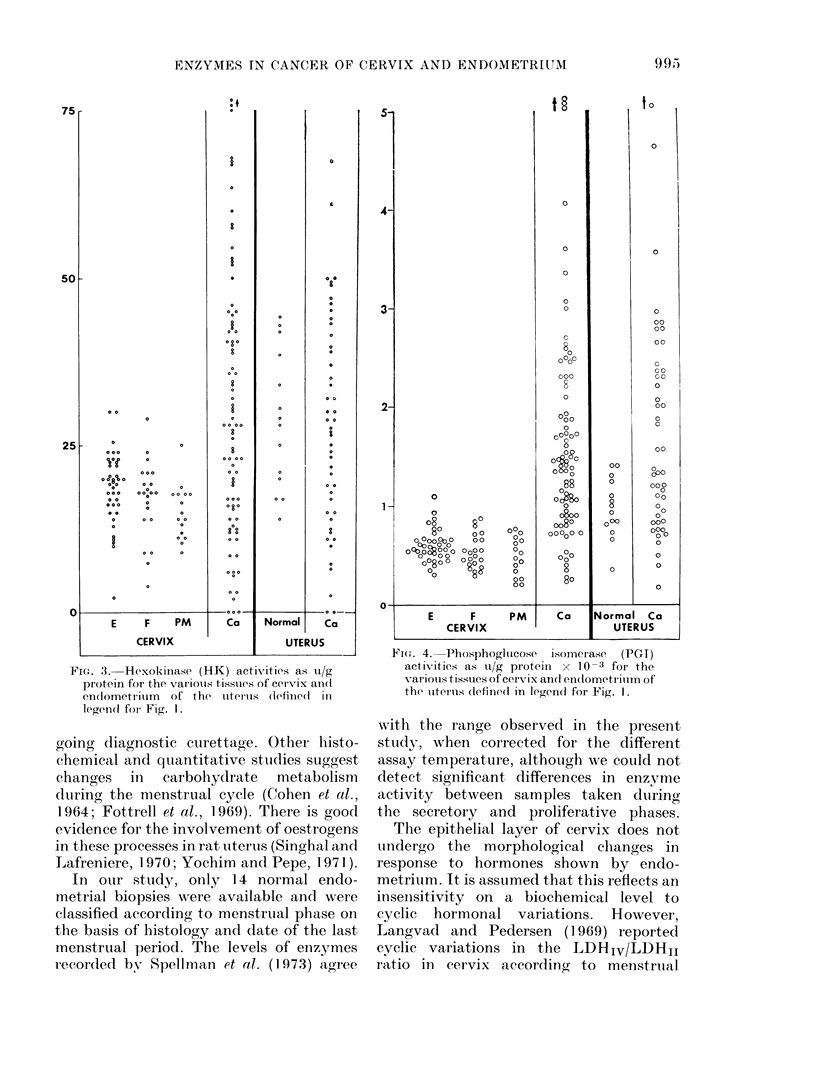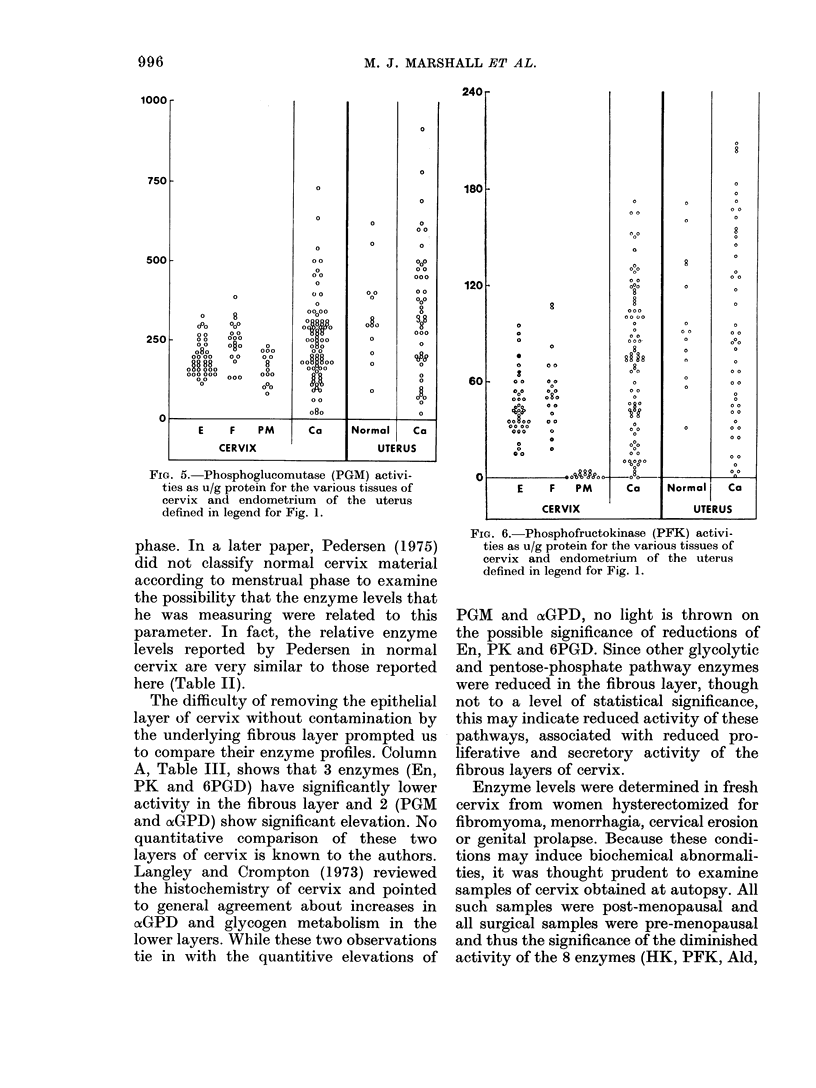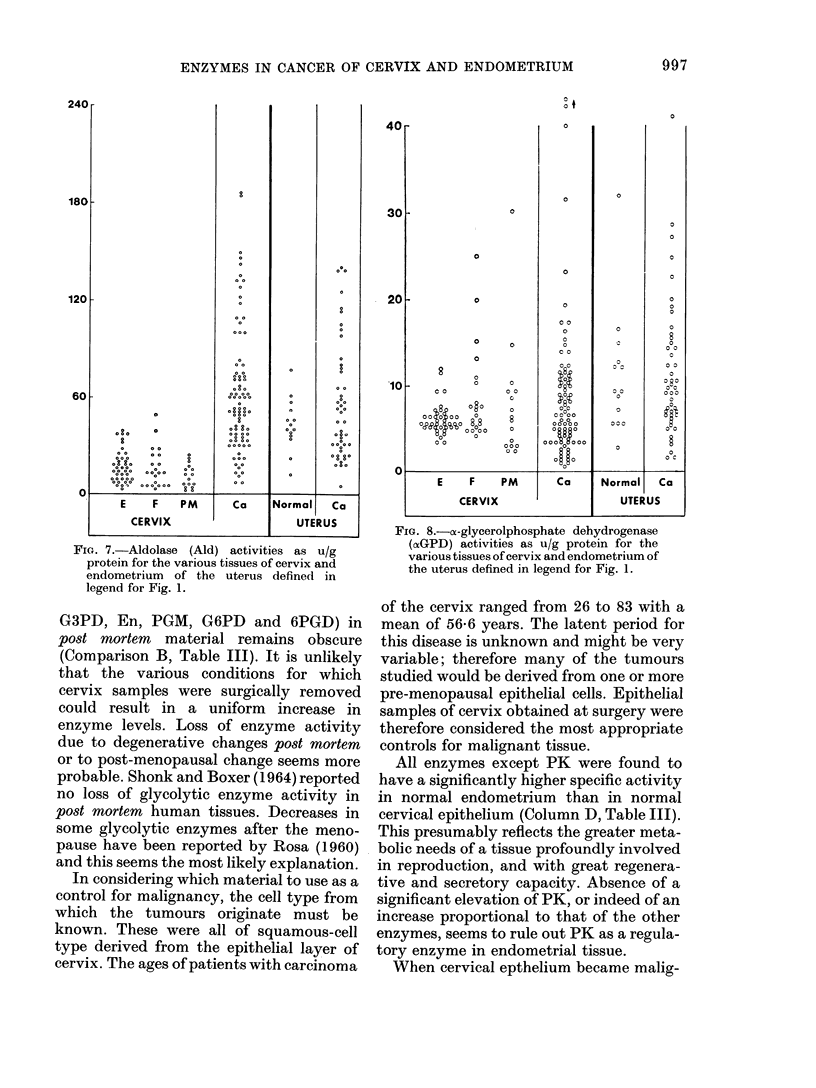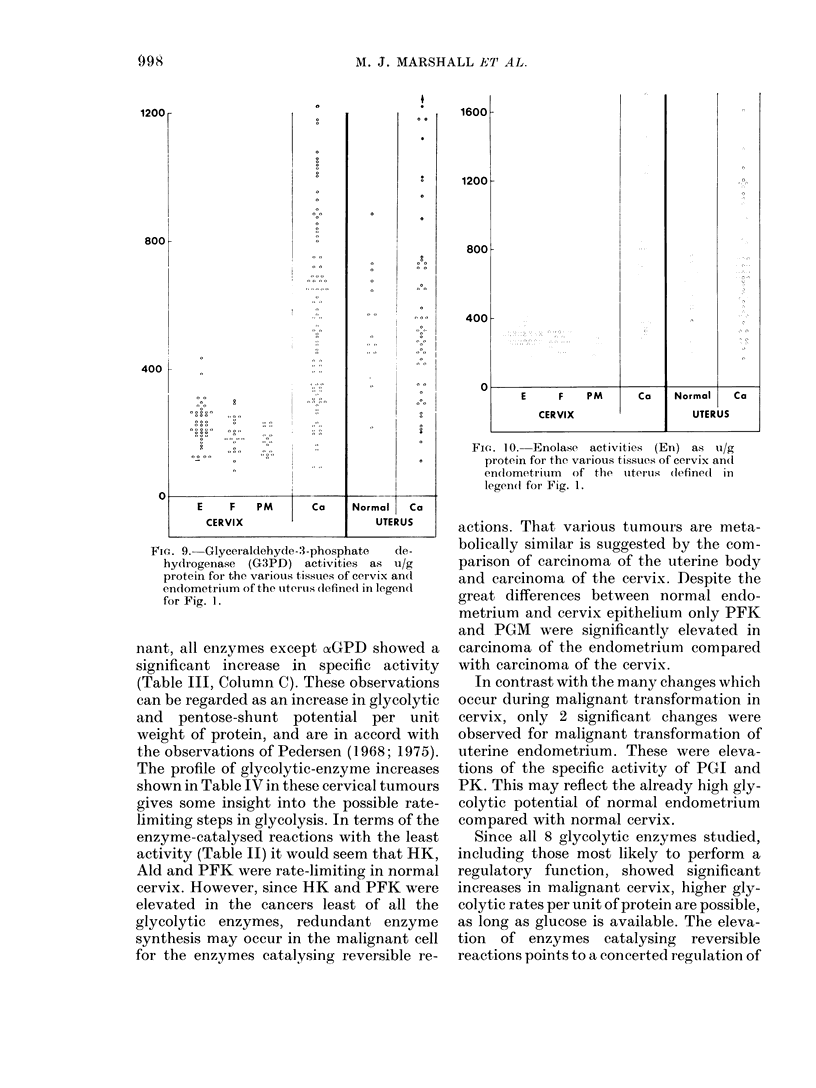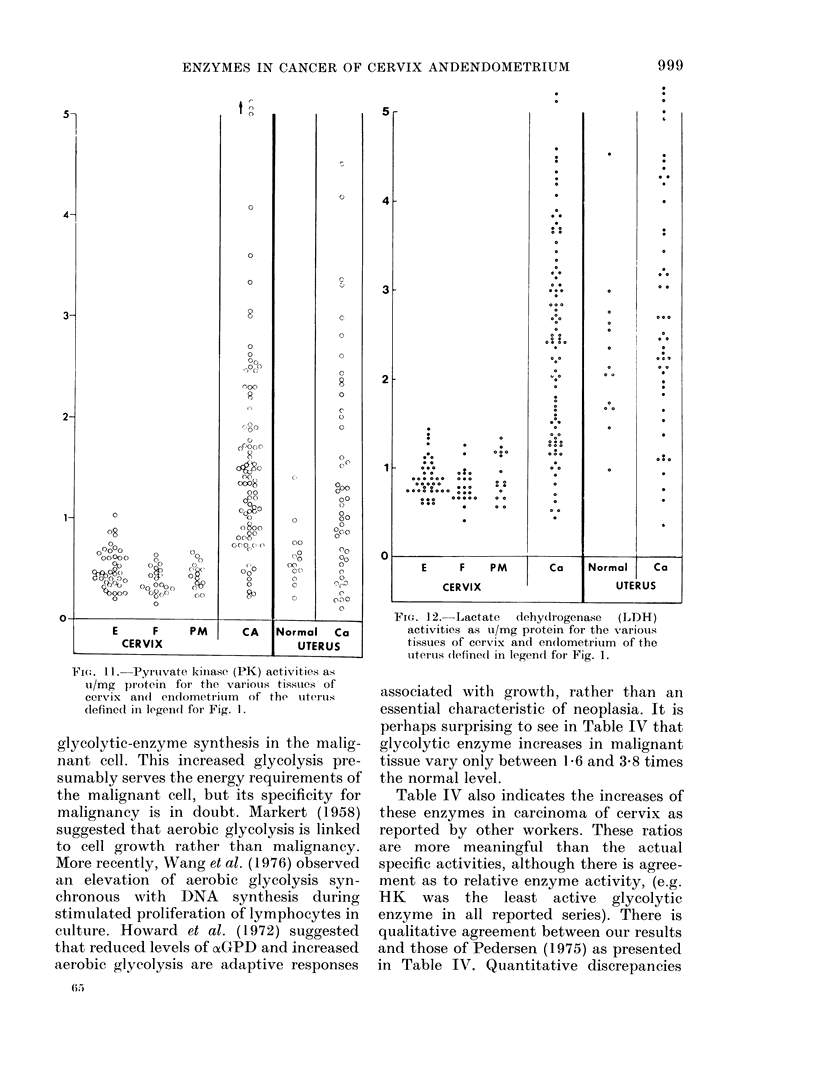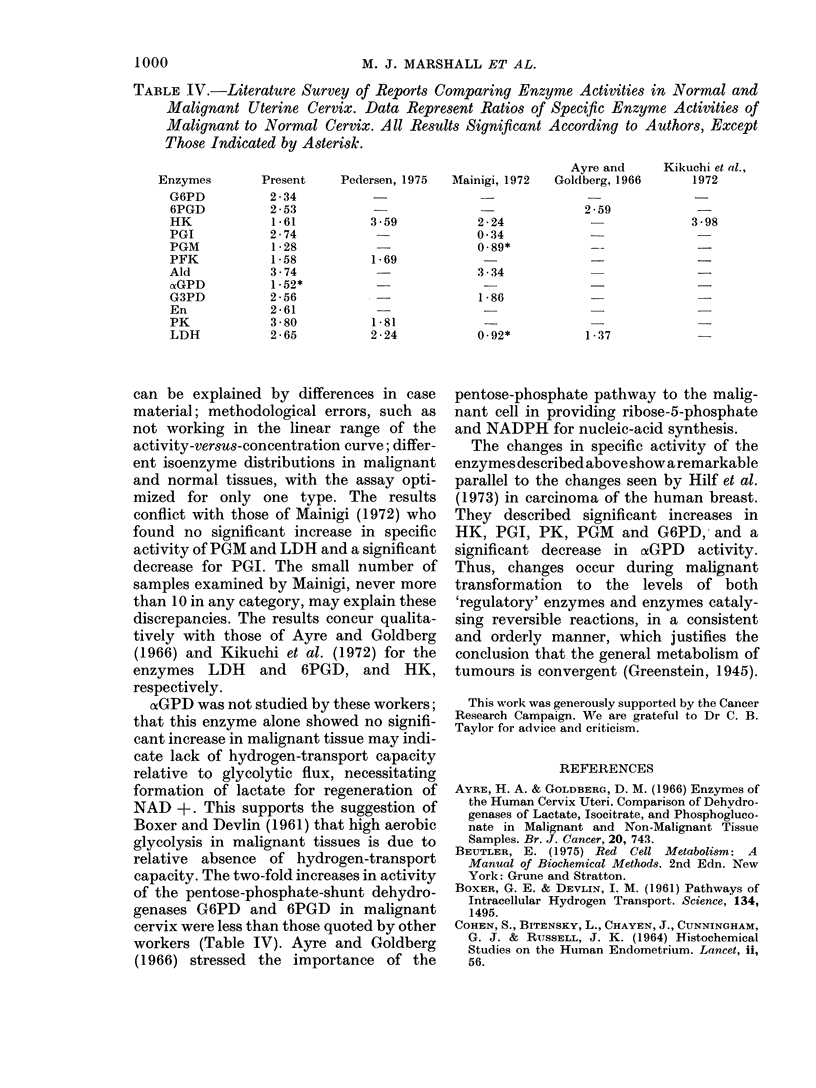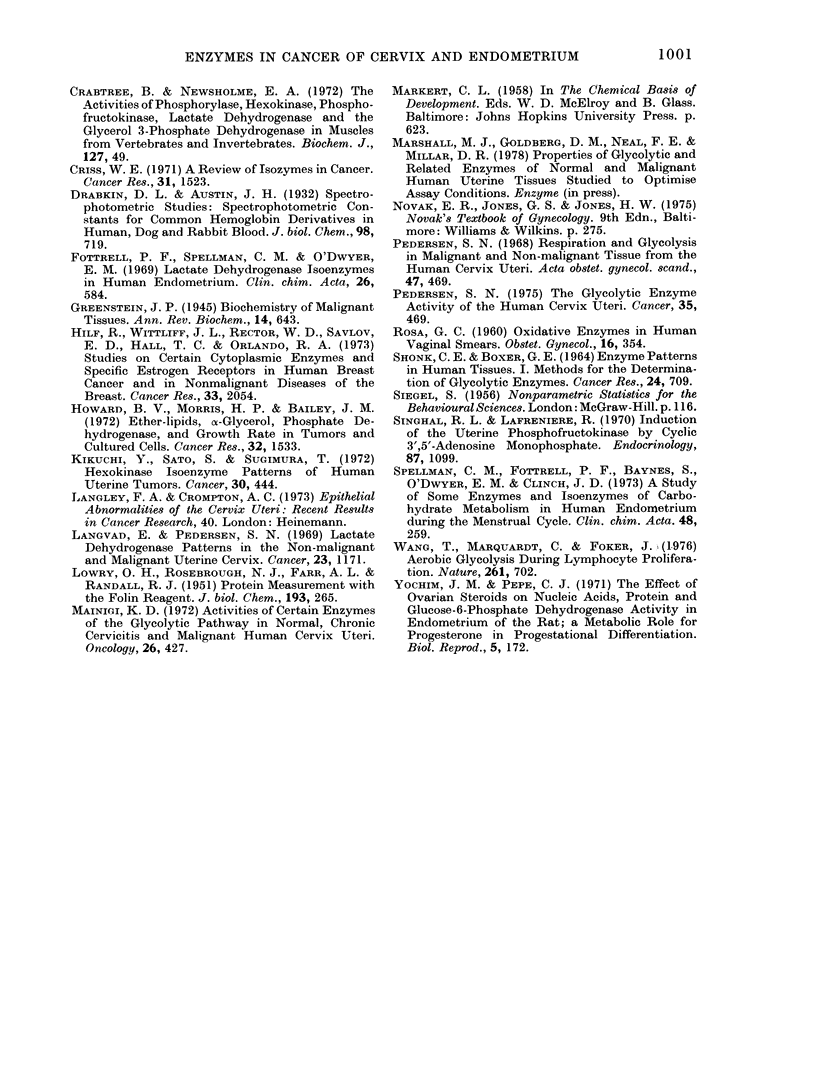# Enzymes of glucose metabolism in carcinoma of the cervix and endometrium of the human uterus.

**DOI:** 10.1038/bjc.1978.144

**Published:** 1978-06

**Authors:** M. J. Marshall, D. M. Goldberg, F. E. Neal, D. R. Millar

## Abstract

Twelve enzymes related to the direct oxidative and glycolytic pathways of glucose metabolism were assayed in 88 cancers of the cervix and 48 cancers of the endometrium of the human uterus, and the activities compared with those obtained from a group of control tissues. Significant increases for all but one of the enzymes studied (alpha-glycerolphosphate dehydrogenase) were found in cancer of the cervix, when compared with normal cervix epithelium. Hexokinase, phoshofructokinase, and aldolase appear to be rate-limiting in normal cervix epithelium; however, since the increase in activity of the first two in cancers was least of all the glycolytic enzymes, redundant enzyme synthesis probably occurs in the malignant cell for the enzymes catalysing reversible reactions. There was virtually no correlation between the activity of any enzyme measured in the cancer sample and histological assessments of the degree of malignancy of the tumour, or the clinical stage of the disease. All enzymes except pyruvate kinase had significantly higher activity in normal endometrium than in normal cervix epithelium, presumably reflecting the greater metabolic requirements of the former tissue. Only phosphoglucose isomerase and pyruvate kinase were significantly higher in endometrial cancer than in normal endometrium, and there were few significant differences between cancers of the cervix and of the endometrium, despite the marked differences in their tissues of origin. These results suggest the changes occur during malignant transformation to the activities of both regulatory enzymes and those catalysing reversible reactions, in a manner justifying the conclusion that the general metabolism of tumours is convergent.


*Br. J. Cancer* (1978) **37**, 990

### ENZYMES OF GLUCOSE METABOLISM IN CARCINOMA OF THE CERVIX AND ENDOMETRIUM OF THE HUMAN UTERUS

M. J. MARSHALL*, D. M. GOLDBERG*, F. E. NEAL† AND D. R. MILLAR‡

*From the *Department of Chemical Pathology, Royal Hospital, †Department of Radiotherapy, Weston Park Hospital, Sheffield; and ‡Department of Gynaecology, Jessop Hospital for Women, Sheffield.*

Received 1 February 1978 Accepted 27 February 1978

**Summary.—Twelve enzymes related to the direct oxidative and glycolytic pathways of glucose metabolism were assayed in 88 cancers of the cervix and 48 cancers of the endometrium of the human uterus, and the activities compared with those obtained from a group of control tissues. Significant increases for all but one of the enzymes studied (α-glycerolphosphate dehydrogenase) were found in cancer of the cervix, when compared with normal cervix epithelium. Hexokinase, phosphofructokinase, and aldolase appear to be rate-limiting in normal cervix epithelium; however, since the increase in activity of the first two in cancers was least of all the glycolytic enzymes, redundant enzyme synthesis probably occurs in the malignant cell for the enzymes catalysing reversible reactions. There was virtually no correlation between the activity of any enzyme measured in the cancer sample and histological assessments of the degree of malignancy of the tumour, or the clinical stage of the disease. All enzymes except pyruvate kinase had significantly higher activity in normal endometrium than in normal cervix epithelium, presumably reflecting the greater metabolic requirements of the former tissue. Only phosphoglucose isomerase and pyruvate kinase were significantly higher in endometrial cancer than in normal endometrium, and there were few significant differences between cancers of the cervix and of the endometrium, despite the marked differences in their tissues of origin. These results suggest the changes occur during malignant transformation to the activities of both regulatory enzymes and those catalysing reversible reactions, in a manner justifying the conclusion that the general metabolism of tumours is convergent.**

HUMAN malignant uterine cervix has a greater capacity for lactate formation than normal uterine cervix (Pedersen, 1968) and this may be a reflection of the elevation of the 5 enzymes studied by Pedersen (1975). Considerable work has been done characterizing enzyme content of transplantable tumours in animals to assess the significance of glycolysis for neoplastic growth (Criss, 1971) and an extension of these findings to human malignancies was sought.

In this paper we report the activities of 12 enzymes in samples of normal and malignant uterine cervix and endometrium to provide a more detailed analysis of the enzymatic steps leading to this increase in glycolytic activity, and to correlate clinical features of the disease and histopathological features of the tumour with the observed biochemical changes.

### MATERIALS AND METHODS

*Source of material.*—Biopsy samples of carcinoma of the cervix and uterine body were obtained prior to radiotherapy from patients under general anaesthesia as part of the normal management. Control tissue, grossly normal cervix and uterine endometrium, was obtained under similar conditions from patients undergoing hysterectomy or diagnostic curettage. Some *post mortem* samples of

Correspondence: Dr D. M. Goldberg, Professor and Chairman, Department of Clinical Biochemistry, University of Toronto; The Hospital for Sick Children, 555 University Avenue, Toronto, Ontario, Canada.

TABLE I.—*Abbreviations and Assay Methods for Enzymes Studied in Present Report*

| Abbreviation | Trivial name | Enzyme Commision name | E.C. number | Substrate concentration (mM) | Activator concentration (mM) | Buffer concentration (M) | pH |
|---|---|---|---|---|---|---|---|
| G6PD | Glucose-6-phosphate dehydrogenase | D-glucose-6-phosphate: NADP oxidoreductase | 1.1.1.49 | G6P(0·6); NADP(0·25) | $MgCl_2$(10·0) | (0·1) Tris HCl | 7·8 |
| 6PGD | 6-phosphogluconate dehydrogenase | 6-phospho-D-gluconate: NADP oxidoreductase (decarboxylating) | 1.1.1.44 | 6PG(0·6); NADP(0·25) | $MgCl_2$(10·0) | (0·1) Tris HCl | 7·8 |
| HK | Hexokinase | ATP: D-Hexose 6-phosphotransferase | 2.7.1.1 | Glucose(2); ATP(4) | $MgCl_2$(8·0) | (0·1) Tris HCl | 7·7 |
| PGI | Phosphoglucose isomerase | D-glucose-6-phosphate ketol isomerase | 5.3.1.9 | F6P(1·66) | KCl(200); $MgCl_2$(5·0) | (0·1) Tris HCl | 8·0 |
| PGM | Phosphoglucomutase | α-D-glucose-1,6-diphosphate: α-D-glucose-1-phosphate phosphotransferase | 2.7.5.1 | GlP(10·0) | Gl,6DP(0·1) | (0·1) Tris HCl | 7·3 |
| PFK | Phosphofructokinase | ATP:D-fructose-6-phosphate 1-phosphotransferase | 2.7.1.11 | ATP(1·0); F6P(3·3) | | (0·05) Tris HCl | 8·0 |
| Ald | Aldolase | Fructose-1,6-diphosphate D-glyceraldehyde-3-phosphate-lyase | 4.1.2.13 | FDP(1·0); FlP(10·0) | | (0·1) Tris HCl | 7·8 |
| αGPD | α-glycerophosphate dehydrogenase | L-glycerol-3-phosphate: NAD oxidoreductase | 1.1.1.8 | DHAP(0·3); NADH(0·1) | | (0·05) Tris HCl | 7·3 |
| TPI | Triosephosphate isomerase | D-glyceraldehyde-3-phosphate ketol-isomerase | 5.3.1.1 | | | | |
| G3PD | Glyceraldehyde-3-phosphate dehydrogenase | D-glyceraldehyde-3-phosphate: NAD oxidoreductase (phosphorylating) | 1.2.1.12 | G3P(0·3); NAD (2·0) | $Na_2HASO_4$(18·0) | (0·1) Tris HCl | 7·5 |
| En | Enolase | 2-phospho-D-glycerate hydrolyase | 4.2.1.11 | 2PG(1·0) | | (0·05) TEA HCl | 6·9 |
| PK | Pyruvate kinase | ATP: pyruvate phosphotransferase | 2.7.1.40 | PEP(1·5); ADP(1·5) | FDP(0·3); $MgCl_2$ (10·0) | (0·1) Tris HCl | 7·5 |
| LDH | Lactate dehydrogenase | L-lactate: NAD oxidoreductase | 1.1.1.27 | Pyruvate (0·75); NADH(0·2) | | (0·05) Phosphate | 6·7 |

The following non-standard abbreviations are used for substrates in this Table and in the text: D-glucose-6-phosphate (G6P); 6-phospho-D-gluconate (6PG); D-fructose-6-phosphate (F6P); D-glucose-1-phosphate (GlP); D-glucose-1,6-diphosphate (Gl,6DP); D-fructose-1,6-diphosphate (FDP); D-fructose-1-phosphate (FlP); dihydroxyacetone phosphate (DHAP); D-glyceraldehyde-3-phosphate (G3P); D( + )2-phosphoglycerate (2PG); phospho-enol-pyruvate (PEP); triethanolamine (TEA).

normal cervix were also obtained within 12h after death from patients with no pelvic disease. The source of the various cervical samples is clearly given in the Results section.

*Sample preparation.*—Biopsy fragments were immediately placed in *isotonic buffer* at 4° C, containing the following in the final concentration stated: 0·05M potassium phosphate, pH 7·4; 0·2M sucrose; 1mM ethylene-diaminetetra-acetic acid; 1mM dithiothreitol. The material was centrifuged at 2000 *g* for 4 min and the supernatant discarded. The residue was then washed with distilled water to lyse any erythrocytes present, and recentrifuged. Fat and necrotic tissue were removed. Cervical epithelium and endometrium were dissected from underlying fibrous layers, but with the former, minor contamination with fibrous tissue occurred, confirmed by microscopic examination. Enzyme levels of the epithelial layer were therefore compared with those of the fibrous stroma, to assess possible contamination effects. All the enzymes studied proved stable in the intact frozen tissue (− 20° C), in agreement with the work of Shonk and Boxer (1964). Samples were frozen until a sufficient number had been obtained; normal and malignant samples were processed in any one batch.

Homogenization was carried out in 10–30 volumes of *isotonic buffer*, using first the Ultra-Turax (Janke and Kunkel, K. G., West Germany) at full speed for 10 sec and then a Potter Elvehjem homogeniser (Sireica, N.Y.) for 2 min at 20,000 rev/min, cooling at half-minute intervals in ice. The homogenate was centrifuged at 4° C and at 70,000 *g* for 30 min. The supernatant was removed and an aliquot taken for protein and haemoglobin estimations (Lowry *et al.*, 1951; Drabkin and Austin, 1932). The remainder was diluted with glycerol (A.R.) to a final concentration of 50% and stored at − 20° C.

All enzymes assayed were linked to the production or utilization of reduced nicotinamide adenine dinucleotide (NADH) or its phosphate (NADPH) using the LKB 8600 Reaction Rate Analyser (LKB-Produkter, Bromma, Sweden). An extinction coefficient of 6·22 $cm^2$/mol at 340 nm was assumed for the reduced coenzymes and used to calculate enzyme activity. A unit (u) was defined as the quantity of enzyme required to produce or consume 1 $\mu$mole of NAD(P)H per minute at 37° C. The assays were derived from Beutler (1975) and Crabtree and Newsholme (1972) but were optimised for normal and malignant cervix, as previously reported (Marshall *et al.*, 1978) and the conditions are shown in Table I. This optimisation involved investigation of the effect of substrate and cofactor concentrations, pH, the upper limit of assay linearity and, where indicated, the concentration of activators. The stability of the 12 enzymes stored in 50% glycerol was monitored over a 2-year period, during which only PFK and G6PD showed a significant decline in activity.

### RESULTS

Table II presents the mean, standard deviation, and number of samples assayed, for each enzyme in each tissue. Both epithelial and subepithelial fibrous stroma were examined in fresh surgical samples of cervix, whereas only the epithelial layer was examined in *post mortem* samples. All normal endometrium samples were obtained at biopsy and were segregated into those obtained at the proliferative and secretory stages of the menstrual cycle. No post-menopausal samples were included. Since there were no significant differences between the two categories and the numbers were small, the data were combined to provide a single group for normal endometrium in Table II. Figures 1–12 show the distribution of samples with respect to the specific activity (u/g protein) of each enzyme.

A summary of the significant differences between groups of samples is presented in Table III. Significance was assessed using Student's *t* test, but where samples were not normally distributed, the non-parametric Mann–Whitney U test (Siegel, 1956) was also used. There was agreement between the two tests in all except the one instance indicated in Table III.

Comparisons within control material can be seen in Columns A, B and D (Table III). Three enzymes (Column A) showed a significant decrease in specific activity from the epithelial to the fibrous layer of surgically removed cervix (En, PK and 6PGD) and two enzymes showed a significant increase (PGM and $\alpha$GPD). The comparison between epithelium obtained

TABLE II.—*Specific Activities (u/g protein) of Enzymes in Normal and Malignant Samples of the Cervix and Endometrium of the Uterus. Data as Mean (s.d.)*

| | Cervix | | | | Endometrium | |
|---|---|---|---|---|---|---|
| | Normal | | | | | |
| Enzyme | Epithelial layer | Fibrous stroma | Necropsy samples | Malignant | Normal | Malignant |
| G6PD | 27·8(13·3) | 23·2(7·38) | 14·1(5·74) | 65·0(49·7) | 44·7(14·2) | 48·9(41·7) |
| 6PGD | 22·2(12·6) | 13·6(4·56) | 11·2(5·61) | 56·3(37·0) | 35·5(11·6) | 49·9(30·9) |
| HK | 18·8(5·36) | 17·0(5·8) | 15·0(4·09) | 30·3(19·6) | 28·9(10·3) | 28·1(16·1) |
| PGI | 608(161) | 547(170) | 497(201) | 1660(1260) | 983(303) | 1760(1200) |
| PGM | 195(53·5) | 243(67·6) | 162(51·9) | 249(131) | 328(141) | 338(198) |
| PFK | 45·1(19·1) | 54·5(22·9) | 1·20(1·73) | 71·5(45·5) | 97·3(37·8) | 91·4(61·4) |
| Ald | 16·7(10·1) | 16·1(11·7) | 11·1(7·2) | 62·4(39·1) | 43·3(16·2) | 55·7(36·5) |
| αGPD | 6·00(1·87) | 8·92(5·28) | 7·80(6·94) | 9·12(10·3) | 11·2(7·16) | 11·4(7·82) |
| G3PD | 229(63) | 197(59) | 171(37) | 586(332) | 548(173) | 570(299) |
| En | 283(65) | 236(55) | 212(40) | 739(386) | 538(170) | 686(348) |
| PK | 503(207) | 385(123) | 435(132) | 1910(2550) | 582(192) | 1440(1040) |
| LDH | 903(212) | 807(196) | 941(241) | 2290(1190) | 2260(859) | 2800(1420) |
| No. of Samples | 41 | 22 | 16 | 88 | 14 | 48 |
| Column (see Table III) | | A | B | C | D | |

TABLE III.—*Significance of Differences (P) in Enzyme Activities (Table II) Between Epithelial Layer of Normal Cervix and Other Uterine Tissues Based on Student's* t *test and Mann–Whitney U test. The Upward Arrow Indicates Significantly Higher Activity in the Tissue Listed in that Column and the Downward Arrow Indicates Significantly Higher Activity in the Epithelial Layer of Normal Cervix. No Value Given Where P >0·05*

| | Tissue compared | | | |
|---|---|---|---|---|
| Enzyme | Column A Cervix (fibrous stroma) | Column B Cervix (necropsy) | Column C Cervix (malignant) | Column D Endometrium (normal) |
| G6PD | — | ↓ 0·001 | ↑ 0·001 | ↑ 0·001 |
| 6PGD | ↓ 0·005 | ↓ 0·005 | ↑ 0·001 | ↑ 0·005 |
| HK | — | ↓ 0·02 | ↑ 0·001 | ↑ 0·001 |
| PGI | — | ↓ 0·05[a] | ↑ 0·001 | ↑ 0·001 |
| PGM | ↑ 0·005 | ↓ 0·05 | ↑ 0·02 | ↑ 0·001 |
| PFK | — | ↓ 0·001 | ↑ 0·001 | ↑ 0·001 |
| Ald | — | ↓ 0·05 | ↑ 0·001 | ↑ 0·001 |
| αGPD | ↑ 0·005 | — | — | ↑ 0·001 |
| G3PD | — | ↓ 0·005 | ↑ 0·001 | ↑ 0·001 |
| En | ↓ 0·01 | ↓ 0·001 | ↑ 0·001 | ↑ 0·001 |
| PK | ↓ 0·02 | — | ↑ 0·001 | — |
| LDH | — | — | ↑ 0·001 | ↑ 0·001 |

[a] U test disagrees with the *P* value obtained from the *t* test at surgery and *post mortem* (Column B) showed that in the latter, 8 enzymes had significantly diminished activity (HK, PFK, Ald, G3PD, En, PGM, G6PD and 6PGD) and none were increased. Column D shows that all enzymes except PK had a significantly higher specific activity in normal uterine endometrium than in normal cervical epithelium.

The changed enzyme levels associated with neoplastic transformation of cervix can be seen in Column C. On progression of cervical epithelium to malignancy, all enzymes except αGPD showed significant increase in specific activity. In contrast, only 2 significant changes were observed in malignant uterine endometrium. These were elevations of the specific activity of PGI ($P < 0.05$) and PK ($P < 0.005$). Despite the great difference between

FIG. 1.—Activities of glucose-6-phosphate dehydrogenase (G6PD) as u/g protein. In this and the following figures, the abbreviations at the foot of each column are as follows: for CERVIX, E = epithelial layer, F = fibrous layer of fresh surgical samples, PM = superficial layer (predominantly epithelial) of *post-mortem* material, Ca = cancer tissue. For UTERUS, Normal = epithelial layer of fresh surgical control endometrium, and Ca = epithelium of endometrial cancers.

FIG. 2.—6-phosphogluconate dehydrogenase (6PGD) activities as u/g protein for the various tissues of cervix and endometrium of the uterus defined in legend for Fig. 1.

normal endometrium and cervical epithelium, only PFK ($P < 0\cdot05$) and PGM ($P < 0\cdot005$) were significantly elevated in carcinoma of the endometrium compared with carcinoma of the cervix.

### *Correlation of enzymes with morphological and clinical findings*

Classification of tumours according to histological type was based on the degree of differentiation, and 3 grades were recognised: well, moderately, and poorly differentiated. The clinical staging of the disease was performed according to the classification of the International Federation of Gynecology and Obstetrics (revised 1973) as outlined in Novak *et al.* (1975). The degree of differentiation was not reflected by the activity of any of the 12 enzymes. Only aldolase showed a significant correlation with clinical staging: paradoxically, highest activities were found in Stage I, lowest in Stages III and IV, and intermediate activities in Stage II.

### DISCUSSION

In choosing appropriate control tissues, one must be alert to variation in the native tissue due to normal physiological processes. The tissues of the uterus are exposed to hormonal stimulation which will affect their functioning up to the menopause. Spellman *et al.* (1973) noted elevations of 7 endometrial enzymes, including HK, PK, G6PD and LDH, during the secretory phase, in a study of 208 patients selected because of regular cycles from 420 under-

FIG. 3.—Hexokinase (HK) activities as u/g protein for the various tissues of cervix and endometrium of the uterus defined in legend for Fig. 1.

FIG. 4.—Phosphoglucose isomerase (PGI) activities as u/g protein $\times 10^{-3}$ for the various tissues of cervix and endometrium of the uterus defined in legend for Fig. 1.

going diagnostic curettage. Other histochemical and quantitative studies suggest changes in carbohydrate metabolism during the menstrual cycle (Cohen *et al.*, 1964; Fottrell *et al.*, 1969). There is good evidence for the involvement of oestrogens in these processes in rat uterus (Singhal and Lafreniere, 1970; Yochim and Pepe, 1971).

In our study, only 14 normal endometrial biopsies were available and were classified according to menstrual phase on the basis of histology and date of the last menstrual period. The levels of enzymes recorded by Spellman *et al.* (1973) agree with the range observed in the present study, when corrected for the different assay temperature, although we could not detect significant differences in enzyme activity between samples taken during the secretory and proliferative phases.

The epithelial layer of cervix does not undergo the morphological changes in response to hormones shown by endometrium. It is assumed that this reflects an insensitivity on a biochemical level to cyclic hormonal variations. However, Langvad and Pedersen (1969) reported cyclic variations in the $LDH_{IV}/LDH_{II}$ ratio in cervix according to menstrual

FIG. 5.—Phosphoglucomutase (PGM) activities as u/g protein for the various tissues of cervix and endometrium of the uterus defined in legend for Fig. 1.

FIG. 6.—Phosphofructokinase (PFK) activities as u/g protein for the various tissues of cervix and endometrium of the uterus defined in legend for Fig. 1.

phase. In a later paper, Pedersen (1975) did not classify normal cervix material according to menstrual phase to examine the possibility that the enzyme levels that he was measuring were related to this parameter. In fact, the relative enzyme levels reported by Pedersen in normal cervix are very similar to those reported here (Table II).

The difficulty of removing the epithelial layer of cervix without contamination by the underlying fibrous layer prompted us to compare their enzyme profiles. Column A, Table III, shows that 3 enzymes (En, PK and 6PGD) have significantly lower activity in the fibrous layer and 2 (PGM and αGPD) show significant elevation. No quantitative comparison of these two layers of cervix is known to the authors. Langley and Crompton (1973) reviewed the histochemistry of cervix and pointed to general agreement about increases in αGPD and glycogen metabolism in the lower layers. While these two observations tie in with the quantitive elevations of PGM and αGPD, no light is thrown on the possible significance of reductions of En, PK and 6PGD. Since other glycolytic and pentose-phosphate pathway enzymes were reduced in the fibrous layer, though not to a level of statistical significance, this may indicate reduced activity of these pathways, associated with reduced proliferative and secretory activity of the fibrous layers of cervix.

Enzyme levels were determined in fresh cervix from women hysterectomized for fibromyoma, menorrhagia, cervical erosion or genital prolapse. Because these conditions may induce biochemical abnormalities, it was thought prudent to examine samples of cervix obtained at autopsy. All such samples were post-menopausal and all surgical samples were pre-menopausal and thus the significance of the diminished activity of the 8 enzymes (HK, PFK, Ald,

FIG. 7.—Aldolase (Ald) activities as u/g protein for the various tissues of cervix and endometrium of the uterus defined in legend for Fig. 1.

FIG. 8.—α-glycerolphosphate dehydrogenase (αGPD) activities as u/g protein for the various tissues of cervix and endometrium of the uterus defined in legend for Fig. 1.

G3PD, En, PGM, G6PD and 6PGD) in *post mortem* material remains obscure (Comparison B, Table III). It is unlikely that the various conditions for which cervix samples were surgically removed could result in a uniform increase in enzyme levels. Loss of enzyme activity due to degenerative changes *post mortem* or to post-menopausal change seems more probable. Shonk and Boxer (1964) reported no loss of glycolytic enzyme activity in *post mortem* human tissues. Decreases in some glycolytic enzymes after the menopause have been reported by Rosa (1960) and this seems the most likely explanation.

In considering which material to use as a control for malignancy, the cell type from which the tumours originate must be known. These were all of squamous-cell type derived from the epithelial layer of cervix. The ages of patients with carcinoma of the cervix ranged from 26 to 83 with a mean of 56·6 years. The latent period for this disease is unknown and might be very variable; therefore many of the tumours studied would be derived from one or more pre-menopausal epithelial cells. Epithelial samples of cervix obtained at surgery were therefore considered the most appropriate controls for malignant tissue.

All enzymes except PK were found to have a significantly higher specific activity in normal endometrium than in normal cervical epithelium (Column D, Table III). This presumably reflects the greater metabolic needs of a tissue profoundly involved in reproduction, and with great regenerative and secretory capacity. Absence of a significant elevation of PK, or indeed of an increase proportional to that of the other enzymes, seems to rule out PK as a regulatory enzyme in endometrial tissue.

When cervical epthelium became malig-

FIG. 9.—Glyceraldehyde-3-phosphate dehydrogenase (G3PD) activities as u/g protein for the various tissues of cervix and endometrium of the uterus defined in legend for Fig. 1.

FIG. 10.—Enolase activities (En) as u/g protein for the various tissues of cervix and endometrium of the uterus defined in legend for Fig. 1.

nant, all enzymes except αGPD showed a significant increase in specific activity (Table III, Column C). These observations can be regarded as an increase in glycolytic and pentose-shunt potential per unit weight of protein, and are in accord with the observations of Pedersen (1968; 1975). The profile of glycolytic-enzyme increases shown in Table IV in these cervical tumours gives some insight into the possible rate-limiting steps in glycolysis. In terms of the enzyme-catalysed reactions with the least activity (Table II) it would seem that HK, Ald and PFK were rate-limiting in normal cervix. However, since HK and PFK were elevated in the cancers least of all the glycolytic enzymes, redundant enzyme synthesis may occur in the malignant cell for the enzymes catalysing reversible reactions. That various tumours are metabolically similar is suggested by the comparison of carcinoma of the uterine body and carcinoma of the cervix. Despite the great differences between normal endometrium and cervix epithelium only PFK and PGM were significantly elevated in carcinoma of the endometrium compared with carcinoma of the cervix.

In contrast with the many changes which occur during malignant transformation in cervix, only 2 significant changes were observed for malignant transformation of uterine endometrium. These were elevations of the specific activity of PGI and PK. This may reflect the already high glycolytic potential of normal endometrium compared with normal cervix.

Since all 8 glycolytic enzymes studied, including those most likely to perform a regulatory function, showed significant increases in malignant cervix, higher glycolytic rates per unit of protein are possible, as long as glucose is available. The elevation of enzymes catalysing reversible reactions points to a concerted regulation of

FIG. 11.—Pyruvate kinase (PK) activities as u/mg protein for the various tissues of cervix and endometrium of the uterus defined in legend for Fig. 1.

FIG. 12.—Lactate dehydrogenase (LDH) activities as u/mg protein for the various tissues of cervix and endometrium of the uterus defined in legend for Fig. 1.

glycolytic-enzyme synthesis in the malignant cell. This increased glycolysis presumably serves the energy requirements of the malignant cell, but its specificity for malignancy is in doubt. Markert (1958) suggested that aerobic glycolysis is linked to cell growth rather than malignancy. More recently, Wang *et al.* (1976) observed an elevation of aerobic glycolysis synchronous with DNA synthesis during stimulated proliferation of lymphocytes in culture. Howard *et al.* (1972) suggested that reduced levels of αGPD and increased aerobic glycolysis are adaptive responses associated with growth, rather than an essential characteristic of neoplasia. It is perhaps surprising to see in Table IV that glycolytic enzyme increases in malignant tissue vary only between 1·6 and 3·8 times the normal level.

Table IV also indicates the increases of these enzymes in carcinoma of cervix as reported by other workers. These ratios are more meaningful than the actual specific activities, although there is agreement as to relative enzyme activity, (e.g. HK was the least active glycolytic enzyme in all reported series). There is qualitative agreement between our results and those of Pedersen (1975) as presented in Table IV. Quantitative discrepancies

TABLE IV.—*Literature Survey of Reports Comparing Enzyme Activities in Normal and Malignant Uterine Cervix. Data Represent Ratios of Specific Enzyme Activities of Malignant to Normal Cervix. All Results Significant According to Authors, Except Those Indicated by Asterisk.*

| Enzymes | Present | Pedersen, 1975 | Mainigi, 1972 | Ayre and Goldberg, 1966 | Kikuchi *et al.*, 1972 |
|---|---|---|---|---|---|
| G6PD | 2·34 | — | — | — | — |
| 6PGD | 2·53 | — | — | 2·59 | — |
| HK | 1·61 | 3·59 | 2·24 | — | 3·98 |
| PGI | 2·74 | — | 0·34 | — | — |
| PGM | 1·28 | — | 0·89* | — | — |
| PFK | 1·58 | 1·69 | — | — | — |
| Ald | 3·74 | — | 3·34 | — | — |
| αGPD | 1·52* | — | — | — | — |
| G3PD | 2·56 | — | 1·86 | — | — |
| En | 2·61 | — | — | — | — |
| PK | 3·80 | 1·81 | — | — | — |
| LDH | 2·65 | 2·24 | 0·92* | 1·37 | — |

can be explained by differences in case material; methodological errors, such as not working in the linear range of the activity-*versus*-concentration curve; different isoenzyme distributions in malignant and normal tissues, with the assay optimized for only one type. The results conflict with those of Mainigi (1972) who found no significant increase in specific activity of PGM and LDH and a significant decrease for PGI. The small number of samples examined by Mainigi, never more than 10 in any category, may explain these discrepancies. The results concur qualitatively with those of Ayre and Goldberg (1966) and Kikuchi *et al.* (1972) for the enzymes LDH and 6PGD, and HK, respectively.

αGPD was not studied by these workers; that this enzyme alone showed no significant increase in malignant tissue may indicate lack of hydrogen-transport capacity relative to glycolytic flux, necessitating formation of lactate for regeneration of NAD +. This supports the suggestion of Boxer and Devlin (1961) that high aerobic glycolysis in malignant tissues is due to relative absence of hydrogen-transport capacity. The two-fold increases in activity of the pentose-phosphate-shunt dehydrogenases G6PD and 6PGD in malignant cervix were less than those quoted by other workers (Table IV). Ayre and Goldberg (1966) stressed the importance of the pentose-phosphate pathway to the malignant cell in providing ribose-5-phosphate and NADPH for nucleic-acid synthesis.

The changes in specific activity of the enzymes described above show a remarkable parallel to the changes seen by Hilf *et al.* (1973) in carcinoma of the human breast. They described significant increases in HK, PGI, PK, PGM and G6PD, and a significant decrease in αGPD activity. Thus, changes occur during malignant transformation to the levels of both 'regulatory' enzymes and enzymes catalysing reversible reactions, in a consistent and orderly manner, which justifies the conclusion that the general metabolism of tumours is convergent (Greenstein, 1945).

This work was generously supported by the Cancer Research Campaign. We are grateful to Dr C. B. Taylor for advice and criticism.

### REFERENCES

AYRE, H. A. & GOLDBERG, D. M. (1966) Enzymes of the Human Cervix Uteri. Comparison of Dehydrogenases of Lactate, Isocitrate, and Phosphogluconate in Malignant and Non-Malignant Tissue Samples. *Br. J. Cancer*, **20**, 743.

BEUTLER, E. (1975) *Red Cell Metabolism: A Manual of Biochemical Methods.* 2nd Edn. New York: Grune and Stratton.

BOXER, G. E. & DEVLIN, I. M. (1961) Pathways of Intracellular Hydrogen Transport. *Science*, **134**, 1495.

COHEN, S., BITENSKY, L., CHAYEN, J., CUNNINGHAM, G. J. & RUSSELL, J. K. (1964) Histochemical Studies on the Human Endometrium. *Lancet*, **ii**, 56.

CRABTREE, B. & NEWSHOLME, E. A. (1972) The Activities of Phosphorylase, Hexokinase, Phosphofructokinase, Lactate Dehydrogenase and the Glycerol 3-Phosphate Dehydrogenase in Muscles from Vertebrates and Invertebrates. *Biochem. J.*, **127**, 49.

CRISS, W. E. (1971) A Review of Isozymes in Cancer. *Cancer Res.*, **31**, 1523.

DRABKIN, D. L. & AUSTIN, J. H. (1932) Spectrophotometric Studies: Spectrophotometric Constants for Common Hemoglobin Derivatives in Human, Dog and Rabbit Blood. *J. biol. Chem.*, **98**, 719.

FOTTRELL, P. F., SPELLMAN, C. M. & O'DWYER, E. M. (1969) Lactate Dehydrogenase Isoenzymes in Human Endometrium. *Clin. chim. Acta*, **26**, 584.

GREENSTEIN, J. P. (1945) Biochemistry of Malignant Tissues. *Ann. Rev. Biochem.*, **14**, 643.

HILF, R., WITTLIFF, J. L., RECTOR, W. D., SAVLOV, E. D., HALL, T. C. & ORLANDO, R. A. (1973) Studies on Certain Cytoplasmic Enzymes and Specific Estrogen Receptors in Human Breast Cancer and in Nonmalignant Diseases of the Breast. *Cancer Res.*, **33**, 2054.

HOWARD, B. V., MORRIS, H. P. & BAILEY, J. M. (1972) Ether-lipids, α-Glycerol, Phosphate Dehydrogenase, and Growth Rate in Tumors and Cultured Cells. *Cancer Res.*, **32**, 1533.

KIKUCHI, Y., SATO, S. & SUGIMURA, T. (1972) Hexokinase Isoenzyme Patterns of Human Uterine Tumors. *Cancer*, **30**, 444.

LANGLEY, F. A. & CROMPTON, A. C. (1973) *Epithelial Abnormalities of the Cervix Uteri: Recent Results in Cancer Research*, 40. London: Heinemann.

LANGVAD, E. & PEDERSEN, S. N. (1969) Lactate Dehydrogenase Patterns in the Non-malignant and Malignant Uterine Cervix. *Cancer*, **23**, 1171.

LOWRY, O. H., ROSEBROUGH, N. J., FARR, A. L. & RANDALL, R. J. (1951) Protein Measurement with the Folin Reagent. *J. biol. Chem.*, **193**, 265.

MAINIGI, K. D. (1972) Activities of Certain Enzymes of the Glycolytic Pathway in Normal, Chronic Cervicitis and Malignant Human Cervix Uteri. *Oncology*, **26**, 427.

MARKERT, C. L. (1958) In *The Chemical Basis of Development*. Eds. W. D. McElroy and B. Glass. Baltimore: Johns Hopkins University Press. p. 623.

MARSHALL, M. J., GOLDBERG, D. M., NEAL, F. E. & MILLAR, D. R. (1978) Properties of Glycolytic and Related Enzymes of Normal and Malignant Human Uterine Tissues Studied to Optimise Assay Conditions. *Enzyme* (in press).

NOVAK, E. R., JONES, G. S. & JONES, H. W. (1975) *Novak's Textbook of Gynecology*. 9th Edn., Baltimore: Williams & Wilkins. p. 275.

PEDERSEN, S. N. (1968) Respiration and Glycolysis in Malignant and Non-malignant Tissue from the Human Cervix Uteri. *Acta obstet. gynecol. scand.*, **47**, 469.

PEDERSEN, S. N. (1975) The Glycolytic Enzyme Activity of the Human Cervix Uteri. *Cancer*, **35**, 469.

ROSA, G. C. (1960) Oxidative Enzymes in Human Vaginal Smears. *Obstet. Gynecol.*, **16**, 354.

SHONK, C. E. & BOXER, G. E. (1964) Enzyme Patterns in Human Tissues. I. Methods for the Determination of Glycolytic Enzymes. *Cancer Res.*, **24**, 709.

SIEGEL, S. (1956) *Nonparametric Statistics for the Behavioural Sciences*. London: McGraw-Hill. p. 116.

SINGHAL, R. L. & LAFRENIERE, R. (1970) Induction of the Uterine Phosphofructokinase by Cyclic 3′,5′-Adenosine Monophosphate. *Endocrinology*, **87**, 1099.

SPELLMAN, C. M., FOTTRELL, P. F., BAYNES, S., O'DWYER, E. M. & CLINCH, J. D. (1973) A Study of Some Enzymes and Isoenzymes of Carbohydrate Metabolism in Human Endometrium during the Menstrual Cycle. *Clin. chim. Acta*. **48**, 259.

WANG, T., MARQUARDT, C. & FOKER, J. (1976) Aerobic Glycolysis During Lymphocyte Proliferation. *Nature*, **261**, 702.

YOCHIM, J. M. & PEPE, C. J. (1971) The Effect of Ovarian Steroids on Nucleic Acids, Protein and Glucose-6-Phosphate Dehydrogenase Activity in Endometrium of the Rat; a Metabolic Role for Progesterone in Progestational Differentiation. *Biol. Reprod.*, **5**, 172.